# Sex-specific innate immunity and ageing in long-lived fresh water turtles (*Kinosternon flavescens*: Kinosternidae)

**DOI:** 10.1186/s12979-023-00335-x

**Published:** 2023-03-09

**Authors:** Anne M. Bronikowski, Ashley R. Hedrick, Greta A. Kutz, Kaitlyn G. Holden, Beth Reinke, John B. Iverson

**Affiliations:** 1 Department of Ecology, Evolution, and Organismal Biology, Iowa State University, Ames, IA 50011 USA; 2 Department of Integrative Biology, Kellogg Biological Station, Michigan State University, 3700 E. Gull Lake Rd., Hickory Corners, MI 49060 USA; 3 Department of Biology, Northeastern Illinois University, Chicago, IL 60625 USA; 4 Department of Biology, Earlham College, Richmond, IN 47374 USA

**Keywords:** Innate immune function, Reptile, Senescence, Sex-specific, Ageing

## Abstract

**Background:**

The progressive deregulation of the immune system with age, termed immunosenescence, has been well studied in mammalian systems, but studies of immune function in long-lived, wild, non-mammalian populations are scarce. In this study we leverage a 38-year mark-recapture study to quantify the relationships among age, sex, survival, reproductive output and the innate immune system in a long-lived reptile, yellow mud turtles (*Kinosternon flavescens;* Testudines; Kinosternidae).

**Methods:**

We estimated rates of survival and age-specific mortality by sex based on mark-recapture data for 1530 adult females and 860 adult males over 38 years of captures. We analyzed bactericidal competence (BC), and two immune responses to foreign red blood cells - natural antibody-mediated haemagglutination (NAbs), and complement-mediated haemolysis ability (Lys) - in 200 adults (102 females; 98 males) that ranged from 7 to 58 years of age captured in May 2018 during their emergence from brumation, and for which reproductive output and long-term mark-recapture data were available.

**Results:**

We found that females are smaller and live longer than males in this population, but the rate of accelerating mortality across adulthood is the same for both sexes. In contrast, males exhibited higher innate immunity than females for all three immune variables we measured. All immune responses also varied inversely with age, indicating immunosenescence. For females that reproduced in the preceding reproductive season, egg mass (and therefore total clutch mass) increased with age,. In addition to immunosenescence of bactericidal competence, females that produced smaller clutches also had lower bactericidal competence.

**Conclusions:**

Contrary to the general vertebrate pattern of lower immune responses in males than females (possibly reflecting the suppressive effects of androgens), we found higher levels of all three immune variables in males. In addition, contrary to previous work that found no evidence of immunosenescence in painted turtles or red-eared slider turtles, we found a decrease in bactericidal competence, lysis ability, and natural antibodies with age in yellow mud turtles.

**Supplementary Information:**

The online version contains supplementary material available at 10.1186/s12979-023-00335-x.

## Background

Ageing in many vertebrate systems is characterized by organismal senescence – declining efficiency and performance of physiological and cellular processes [1] leading to declining age-specific survival and fertility with advancing age [2]. Studies of ageing in wild populations of vertebrates have often focused on quantifying age-related changes in fecundity and mortality [3], but less often on physiological mechanisms that may contribute to such demographic ageing (e.g., [4–6]). One such candidate physiological mechanism is immune function, which plays a critical role in survival. Reduced immune function has been shown to negatively impact survival and reproduction [6–8]. The progressive deregulation of the immune system with age, termed immunosenescence, has been well studied in humans for both innate immunity (whose dysregulation with age can lead to chronic inflammation [9]), and acquired immunity, where the best studied changes are an increase in memory T cells and decrease in naïve T cells with advancing age (but here too, immunosenescence remains enigmatic [10]). However, age-specific changes in the immune system of long-lived, wild, non-mammalian populations are not well described in the literature, and studies focusing on reptile immunosenescence are even more rare (reviewed in [8]).

Organismal senescence, which gives rise to demographic senescence in populations, evolved in the wild. Thus, studies on natural populations are well positioned to reveal evolutionarily conserved senescent processes that are possible constraints for how ageing occurs. At the same time, studying organismal and demographic senescence in the wild provides realistic ecological contexts and reveals the extent to which ageing mechanisms are flexible. In particular, reptiles have many features that recommend them for studies of the mechanisms of senescence, including immunosenescence. First, they are the sister clade to mammals, which together form the monophyletic clade of amniotes; immune function and cell signaling pathways are highly conserved across amniotes (reviewed in [11]). Second, reptiles exhibit a number of phenotypes that allow the study of senescence across a range of physiological and morphological contexts, which is not possible in most mammalian species (e.g., hypoxia resistance, freeze-tolerance, venom, armor, heat tolerance, reviewed in [8]). Excepting birds, reptiles are poikilothermic, and many continue growing as adults (e.g., [12] but see [13]) and increasing their fecundity with advancing age ([14–17], reviewed in [8]). Furthermore, many reptile lineages have unique morphological features. Examples of the latter include protective phenotypes - such as external ribcages in turtles and venom in some snakes.

Recent phylogenetic analyses of rates of mortality ageing showed strong support for the hypothesis that reptile lineages with protective phenotypes had longer lives and slower ageing than similarly sized mammals, particularly across turtles [18], (see also [19, 20]). Indeed, across 300 species of wild-sampled tetrapods [18], turtles were unique in that the entire lineage was characterized by species with nearly negligible mortality ageing and long lifespans – a finding mirrored in captivity [21]. Given that growth (and therefore, fecundity) may continue over the adult lifespan, selection against deleterious mutations with late-age phenotypes may be stronger in older ages in turtles and other reptiles with these characteristics relative to mammals (as in [22]). These traits suggest that immunosenescence may not manifest in the same manner in reptiles when compared to other amniote vertebrates, such as mammals or birds, and may even be absent due to selective pressures on maintaining immune function with increased reproduction (e.g., [5]). Here we focus on expanding the comparative landscape of ageing studies in turtles to promote their use as models of slow ageing.

The immune system of turtles, like other amniotes, includes innate and acquired components (reviewed in [23]), but recent analyses suggest that reptiles may rely more heavily on innate defenses relative to mammals and birds [24]. Specifically, although reptiles have B and T cells associated with adaptive immunity, the traditional adaptive rapid response upon secondary exposure to pathogens may rely more heavily on innate immunity [24]. There is a growing literature on age-associated patterns of innate immunity in ectothermic reptiles [4, 25–27], which affords a first line of defense against foreign pathogens. At the same time, the study of sex-differences in both organismal senescence and demographic ageing has expanded to better phylogenetic coverage, including reptiles [28]. Importantly, these studies are possible in wild populations only with the ability to accurately age individuals, which requires long-term study for long-lived species ([29, 30], reviewed in [31]). Thus we add to this literature by investigating aspects of sex- and age-specificity of innate immunity, survival, female reproduction, and relationships among them in a long-lived reptile. We leverage a 38-year mark-recapture study of known-age yellow mud turtles (*Kinosternon flavescens*) to quantify the relationships among age, sex, survival, and reproductive output on the innate immune system as an integrative approach to understanding immunosenescence. Like other turtles, this species was found to have slow ageing when the sexes are considered together [18]. Specifically, we assessed three innate immune measures – circulating natural antibodies, complement-mediated lysis ability, and bactericidal competence of plasma – in our long-term study population in Nebraska (USA). In accordance with the existing paradigm of innate immunosenescence, we predicted that measures of innate immunity would decline with advancing age, despite some evidence to the contrary [5, 32].

## Results

Two hundred animals were sampled in 2018 for immune variables (*N* = 98 males, *N* = 102 females) and these observations comprise our immune data. Of these 102 females, 85 reproduced in 2017 and these comprise our female reproductive data. Finally, for the analysis of mortality senescence in the population as a whole, we used our long-term database of *N* = 2380 unique known-age individuals (*N* = 860 males, *N* = 1530 females). In our sample of immune data individuals (200 adults), age was highly correlated with body size; older animals were larger in both mass and length (e.g., body mass, *r* = 0.58, *Pr.* < 0.0001; plastron length, *r* = 0.60, *Pr.* < 0.0001). Therefore body size was not included in models where age was an explanatory factor.

In the immune data individuals, adult females were smaller than males and tended to be older (Table 1, Fig. S2), a result that is mirrored in the population as a whole (see Fig. 5 in [33]). Because adult females in our immune sample were significantly older than males, we z-transformed age for all analyses of innate immune function to dissociate the confound of age and sex. This allowed for a comparison of relatively old and young adult males and females.

**Table 1:** Size, Age, and Mortality Aging in yellow mud turtles

	Females	Males
**Carapace length (mm)** **(Range)**	100.4 ± 5.5 (89.3–112.1)	115.3 ± 5.9 (102.7–128.1)
**Age (Yr)** **(Range)**	27.5 ± 11.6 (11–58)	21.0 ± 10.2 (9–55)
**Median Lifespan (Yr)** **(Age** ***x,*** **50% of adults alive)**	22.6	19.4
**Maximum Lifespan (Yr)** **(Age** ***x,*** **5% of adults alive)**	42.4	35.6
**Initial adult mortality rate (IMR)** **(95% Credible Interval)**	0.05 (0.04–0.053)	0.07 (0.06–0.08)
**Rate of Ageing (RoA)** **(95% Credible Interval)**	0.041 (0.003–0.05)	0.043 (0.003–0.056)
**Mortality rate doubling time (Yrs)**	16.1	16.9

Mean adult body size and age ± 1 SD for the *N* = 102 females and *N* = 98 males included in the immune measures (above the bold line). Below the bold line, for the entire sample of *N* = 1530 female and *N* = 860 marked animals in this population, median and maximum adult lifespan (age *x* at which 50 and 5% adults remain alive, respectively); ageing parameters obtained by fitting Gompertz accelerating mortality model of the age-specific mortalities: u_x_ = *A*e^*b*x^ where *A* is the initial adult mortality rate (IMR) modeled at age 11 years for both sexes and *b* is the rate of accelerating mortality (Rate of Ageing, RoA); and Mortality rate doubling time (Ln(2)/*b*)

**Fig. 1:**
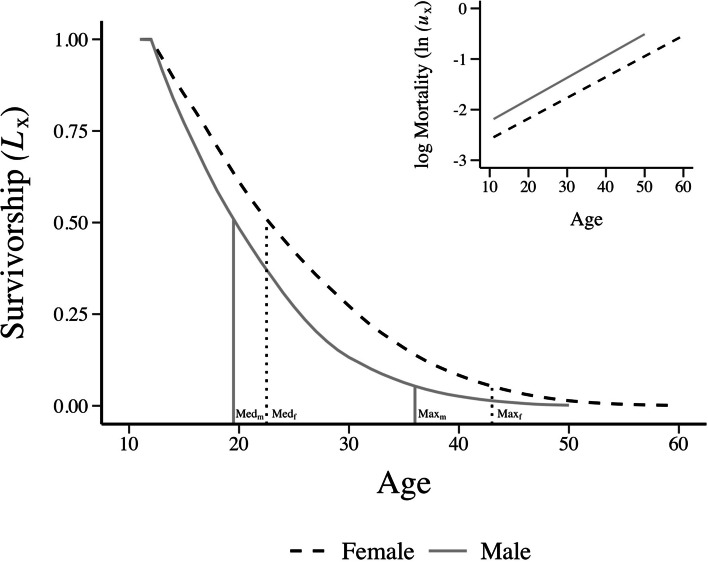
Survivorship and age-specific mortality in adult male and female *K. flavescens*. Median (50% alive) and maximum (5% alive) lifespan is greater in adult female (black dashed lines) yellow mud turtles than in adult males (grey solid lines). Y-axis is survivorship Lx for adults, starting at age 11. Lines dropping to the x-axis show the sex-specific median and maximum lifespans in chronological age. The inset shows the Gompertz models of accelerating mortality across adulthood; the initial adult mortality for males is significantly higher than for females (elevation of lines), with equivalent increases (slopes of lines) (Table 1). Y-axis is natural logarithm of age-specific mortality from the fitted Gompertz acceleration model

In the population as a whole, females had greater median and maximum adult lifespans (ages at which 50 and 95% of adults had died). Adult male and female turtles both exhibited age-related morality increases, indicating ageing. Initial adult mortality rate was higher in males than females, but rates of mortality acceleration were equivalent (Table 1, Fig. 1).

**Fig. 2:**
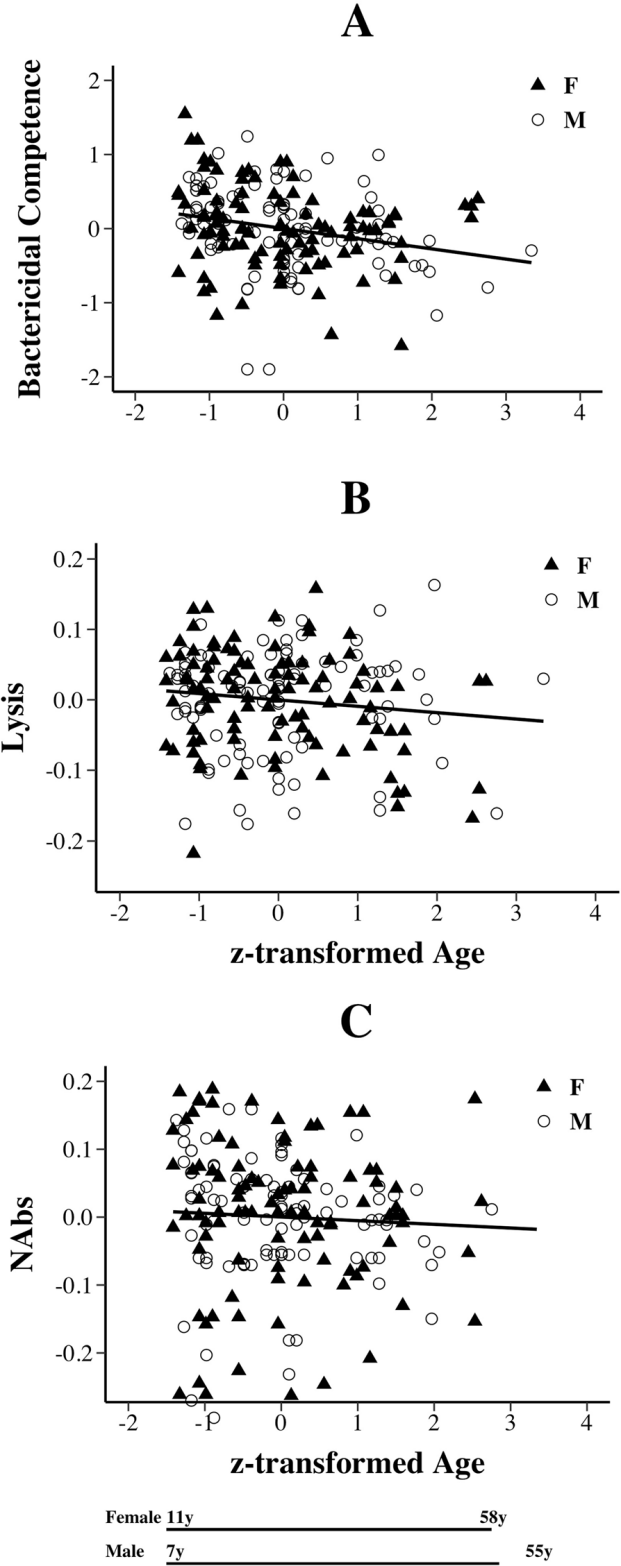
Innate Immunity declines with age in adult *K. flavescens*. **A** Bactericidal Competence and **B** Complement-mediated lysis decreased with age similarly for adult male and female yellow mud turtles (See Table 2 for statistical output). **C** Natural Antibodies (NAbs) showed the same trend. In all panels, the Y-axis represents residuals from linear models with age removed from the model (Table 2). The X-axis is z-transformed age with inset lines to indicate age-in-years for male and female adult turtles. Regressions: BC residuals = − 0.138(zAge), *R*^2^ = 0.06; Lysis residuals = − 0.01(zAge), *R*^2^ = 0.02; NAbs residuals = − 0.01(zAge), *R*^2^ = 0.01

**Fig. 3:**
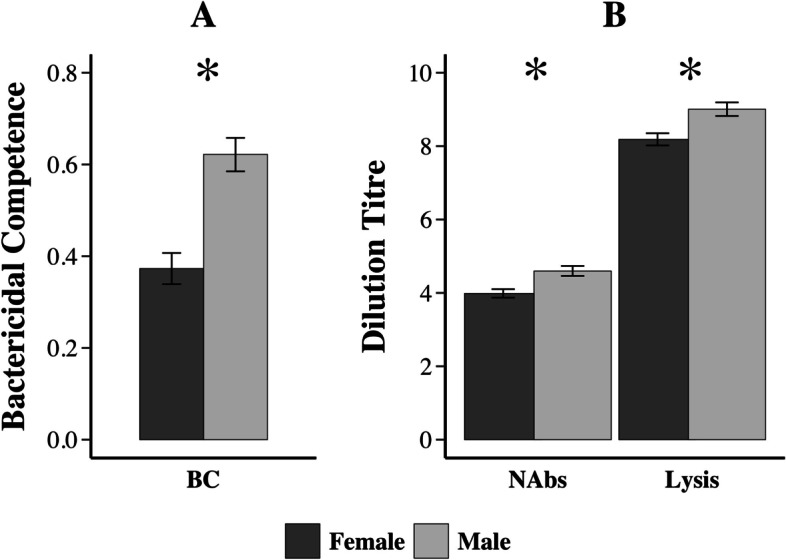
Innate Immunity is significantly higher in male than female adult *K. flavescens*. Back transformed least-square means ±1 SE for **A**) bactericidal competence (y-axis is proportion of *E. coli* colonies killed) and **B**) natural antibodies (y-axis is maximum dilution titre at which haemagglutination of rabbit red blood cells occurs) and complement-mediated lysis (maximum dilution titre at which lysis of rabbit red blood cells occurs). Asterisks represent a significant sex difference for a given immune measure. (See Table 2 for statistical output). LS means: BC = 0.37, 0.62; NAbs = 4.0, 4.6; Lys = 8.2, 9.0

In our immune subsample of individuals, the three measures of innate immune function were significantly (positively) correlated with each other (natural antibody-mediated haemagglutination, complement-mediated haemolysis, and bactericidal competence, Table S1). But because correlations were weak (all |*r*| <  0.28), we analyzed each variable separately. For all three variables, batch represents reagents used. For bactericidal competence, five individual lyophilized pellets of *Escherichia coli* were used to generate control plates across the experiment; as control plates decreased in their colony count, a fresh control solution was made. For natural antibodies and cell lysis capability, two separate bottles of rabbit red blood cells were used sequentially. Significant variation among *E. coli* pellets and individual rabbits (that generate the red blood cells) is expected, and we controlled for it here as a batch effect in our models. All three immune variables declined with advancing age (BC and Lysis, *Pr* <  0.05; Nabs, *Pr* = 0.08, Table 2, Fig. 2) and were higher in males than females (Table 2, Fig. 3).

**Table 2:** Analysis of variance for immune variables for adult male and female yellow mud turtles

Source of Variation	Asin(BC)	Log(NAbs)	Log(Lys)
zAge			
*F* (d.f._n_, d.f._d_)	13.98 _1, 187_	3.01 _1, 191_	3.61 _1, 191_
*P*_r_ > F	**0.0002**	**0.08**	**0.05**
	**Old < Young**	**Old < Young**	**Old < Young**
	(Fig. 2A)	(Fig. 2C)	(Fig. 2B)
Sex			
*F* (d.f._n_, d.f._d_)	38.97 _1, 187_	35.89 _1, 191_	15.49 _1, 191_
*P*_r_ > F	**< 0.0001**	**< 0.0001**	**< 0.0001**
	**M > F**	**M > F**	**M > F**
	(Fig. 3A)	(Fig. 3B)	(Fig. 3B)
zAge x Sex			
*F* (d.f._n_, d.f._d_)	0.80 _1, 187_	0.24 _1, 191_	0.41 _1, 191_
*P*_r_ > F	0.37	0.62	0.26
Beach			
*F* (d.f._n_, d.f._d_)	1.86 _2, 187_	0.02 _2, 191_	1.34 _2, 191_
*P*_r_ > F	0.16	0.98	0.26
Batch			
*F* (d.f._n_, d.f._d_)	15.81 _4, 187_	189 _1, 191_	7.06 _1, 191_
*P*_r_ > F	**< 0.0001**	**< 0.0001**	**0.009**

Bactericidal Competence was Arcsine transformed (Asin(BC)). Titres of natural antibody-mediated haemagglutination (NAbs) and complement-mediated haemolysis (Lys) were both Log10 transformed (Log(NAbs), Log(Lys)). (See text for details.) *P*-values less than 0.10 are boldface

**Fig. 4:**
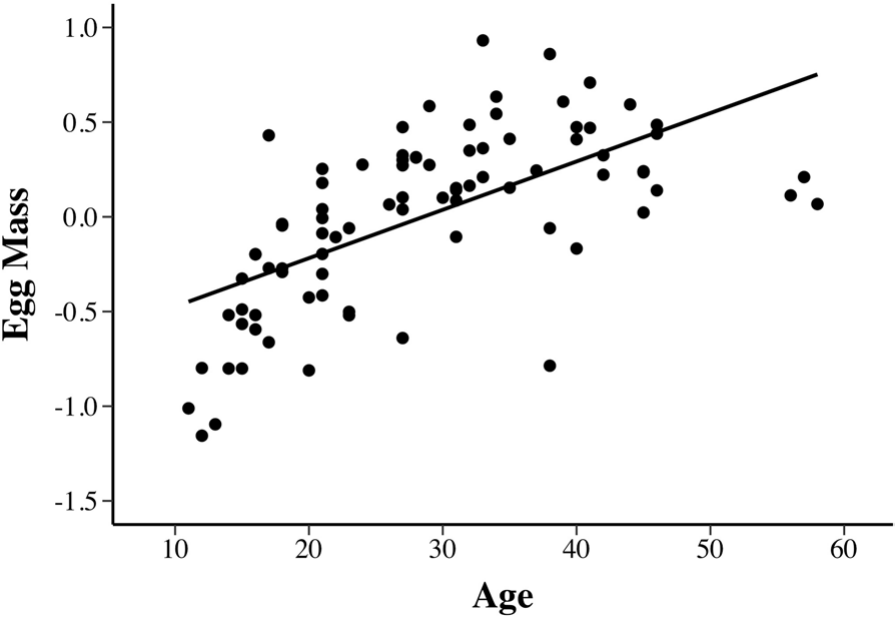
Egg mass, not clutch size, increases with age in reproducing female *K. flavescens*. The Y-axis represents residuals from the linear model in Table 3 with age removed from the model. Egg mass residuals = 0.025(Age) – 0.73, *R*^2^ = 0.40

For the 85 females in our data for which we had both most-recent reproductive output and immune measures, clutch size did not change with age, whereas egg mass increased with age (Tables 3, S2, Fig. 4). Because clutch size did not change with age, a third measure of reproductive effort, total clutch mass followed the identical trend as egg mass and was not considered further. Interestingly, the three females greater than 50 years old had lower age-corrected egg mass (age > 50 years) than females in the next lowest age interval (*N* = 13 age 40–50 years of age (*t* = 2.45, *Pr.* = 0.014)) suggesting the possibility of senescence in reproductive output in very old females (see Fig. 4).

**Table 3:** Analysis of immune variables and reproductive effort for reproductive female yellow mud turtles

Source of Variation	Asin(BC)	Log(NAbs)	Log(Lys)	Egg Mass
Age				
*F* (d.f._n_, d.f._d_)	5.10 _1,78_	0.81 _1, 81_	4.06 _1, 81_	54.7 _1, 88_
*P*_r_ > F	**0.027**	0.37	**0.047**	**< 0.0001**
	**Old < Young**		**Old < Young**	**Old > Young**
	(Fig. 2A)		(Fig. 2B)	(Fig. 4)
Clutch Size				
*F* (d.f._n_, d.f._d_)	4.79 _1,78_	0.52 _1,81_	0.29 _1,81_	2.44 _1, 82_
*P*_r_ > F	**0.031**	0.47	0.59	0.12
	**Large > Small**			
	(Fig. S1)			
Batch				
*F* (d.f._n_, d.f._d_)	6.57 _4, 78_	110 _1, 81_	2.21 _1, 81_	–
*P*_r_ > F	**0.0001**	**< 0.0001**	0.14	–

Analysis of variance of immune variables and egg mass for reproducing females as a function of age and clutch size, Immune Variables are transformed as in Table 2. (See text for details)

For these 85 reproductive females, older females followed the same trend observed for all adults: reduced bactericidal competence and complement-mediated haemolysis with age (Table 3, see Fig. 1). In addition, females with larger clutches - irrespective of age - had higher bactericidal competence than those with smaller clutches (Fig. S1). In contrast to clutch size, we found no effects of egg mass on immune function (data not shown).

## Discussion

### Mortality and reproductive senescence

Female yellow mud turtles live longer than males in this population; however, they age at similar rates (Fig. 1) and age-specific mortality increases with age in both sexes (Fig. 1), albeit slowly. These data demonstrate slow mortality senescence in this species, but we found no evidence of reproductive senescence in our female samples. Small acceleration in adult age-specific mortality in turtles have been reported in the large tetrapod phylogenetic study [18] as well as in individual long-term studies for giant tortoises [34], painted turtles ([35, 36] but see [37, 38] and species of hinge-back tortoises [39]); however, in other turtle species, mortality often remains constant with advancing adult age [40], or decreases with age [3, 15, 41]. Across captive populations of turtles (e.g., zoo records) da Silva et al. [21] found that most turtle species exhibited slow or negligible declines in mortality with age. Female reproductive ageing (i.e., declines in age-specific reproductive output) is not seen in turtle species thus far (painted turtles [30], Blanding’s turtles [15, 41], desert tortoises [3], and box turtles [42]). Warner and colleagues [30] did note decreased painted turtle hatchling survival from the oldest female painted turtles, despite no decrease in reproductive output. In addition, many reproductive studies of turtles have demonstrated positive correlations between clutch size, egg size and/or clutch mass with body size in turtles ([43]; reviewed [44]), where body size is often positively associated with age in turtles (e.g., the present study, [17, 32], but see [41]). These data suggest that reproductive senescence rarely occurs in turtles, if at all.

### Sex-specific longevity and ageing

Studies of sex differences in organismal and demographic senescence remain rare in non-mammalian species. There are likely two reasons for this: initial reports of demography on wild populations often pool data for sufficient power to estimate mortality acceleration; and in many species the sex of juveniles is unknown due to a lack of external genitalia, and must rely on the development of secondary sexual phenotypes to assign sex in newly mature animals. In a review of captive longevity data, da Silva and colleagues [21] reported that average adult male life expectancy exceeded that of females by about 20%, contrary to our results for yellow mud turtles. However, greater longevity in females in wild populations of turtles appears to be the pattern (painted turtles [28, 45]), spotted turtles [46, 47], wood turtles [48], map turtles [49] cooters [50], box turtles [51], and red-eared sliders [52]). In contrast, we are aware of only two studies that have reported that male turtles outlive females in the wild (gopher tortoises [53] but see [54], and a population of painted turtles [36]. Further data are needed, but these field data contrast with the captive records summarized by [21], and suggest a possible sex by environment interaction related to longevity and ageing.

### Sex-specific immune function

We found higher levels of immune function in males than females, the opposite of the general pattern in vertebrates [55, 56], and presumed to be related at least in part to immune system suppression by androgens [57, 58]. Many species of turtles are appropriate models for this question because they lack sex chromosomes, and therefore the sexes have identical genome architecture at conception. They instead have temperature-dependent sex determination (TSD) whereby sex is determined by the thermal environment experienced during the critical period of embryonic development, removing any confound between sex-specific genomes and sex-specific sexual development [59]. López-Pérez and colleagues [60] found no difference in bactericidal competence between males and females in a species of musk turtle (another kinosternid turtle with TSD). Likewise, Zimmerman and colleagues [32, 61, 62] found no sex difference in measures of innate immunity in red-eared slider turtles (also with TSD). In contrast, in painted turtles [5] – another species with TSD –, higher complement-mediated haemolysis ability was reported in males, and higher natural antibody titres in females, with no sex effect on bactericidal competence. In addition, Freedberg and colleagues [63] found that cool, male-producing incubation temperatures in another TSD turtle enhanced immunocompetency in males more than females, but found no such effects in a closely related congener. Although these preliminary data seem to suggest that turtles may not follow the general vertebrate pattern of lower immune responses in males, further research is clearly needed. Following ecoimmunology theory that posits associations between immune defenses and life-history strategies [64, 65], we would expect the longer-lived sex to invest more heavily in acquired relative to innate immunity [66]; limited data on sex-specific longevities precludes a comprehensive test across species.

Behavioral differences and activity patterns may subject female yellow mud turtles to greater infection pressures than males, which may suppress female immune function [55, 56]. Our study species has an unusual annual activity cycle (see Methods). At our study site adult males emerge from brumation an average of 5 days before females in the spring [Iverson and Greene, unpublished data], remain active in the water in June during the extended nesting forays by females, and on average move to summer estivation later than females [67]. Hence, the annual activity period of this species is unusually short, with a typical male being active in the water perhaps 25% longer than a typical female. However, it is not clear why the shorter activity season of females would expose them to greater immune system pressures. As well, reproductive activity differs between males and females, with mating and fertilization occurring in the water column, after which females must emerge from the water to land to deposit their eggs (whereas males have no such mandatory foray onto land) [67].

Reptilian circulating steroid concentrations are highly seasonal (e.g., [68, 69]), and thus the pattern we observed of higher immune function in males might be attributed to sampling only during spring emergence – i.e., very early in the season. Seasonal variation in turtle androgens have been reasonably well studied, and circulating testosterone levels for male turtles are generally very low in the early spring (review in [69]), whereas it peaks in the spring for females as they prepare for ovulation [69–74]. Testosterone is known to suppress immune function ([57], but see [58]) and this might explain the lower immune responses in females compared to males. However, estradiol is known to enhance immune function [23], and it is generally elevated in females in the early spring, followed by a peak soon afterward in association with ovulation and oviposition ([75], reviewed in [69]). Thus, based on our early spring sampling, estradiol and testosterone patterns are unlikely explanations for the lower immune responses observed in our females. Unfortunately, we lack data for circulating hormone levels in our study species, but given the general patterns just described, the impact of circulating steroids on immune function is still not clear.

### Female reproduction and immune function

We found no evidence for an immune cost to female reproduction. Neither clutch size (Table 3) nor egg mass (data not shown) negatively impacted any immune variable. Indeed, females that laid larger clutches of eggs had higher bactericidal capability (Fig. S1). Life history theory predicts that individuals must allocate resources into traits such as those that support self-maintenance and reproduction in order to maximize fitness [76]. However, reproduction represents a significant energetic cost to females and thus resources allocated to reproduction may come at the expense of other traits [77]. Females investing heavily in current reproduction may thus be expected to experience trade-offs with current immune effectiveness [78]. This trade-off between reproduction and immune function has been examined in other reptiles with mixed results. Gravid western terrestrial garter snakes (*Thamnophis elegans*) displayed lower T-lymphocyte proliferative ability in response to a mitogen relative to non-gravid conspecifics but exhibited no difference in BC between reproductive states [27]. In contrast, gravid pygmy rattlesnakes (*Sistrurus milarius*) displayed decreased BC ability relative to non-gravid snakes [79]. Measures of innate immunity between reproductive states in painted turtles (*Chrysemys picta*) were age dependent [5]. Specifically, younger females that had large clutches exhibited greater lysis ability than older females with similarly large clutch sizes. However, this same study found increased BC in older females which is in contrast to the findings in this study of decreasing immune function with advancing age. Additionally, irrespective of age, we found that larger clutch sizes were correlated with higher BC and that there was no effect of reproduction on natural antibody production or lysis ability. These results suggest that trade-offs between immune function and reproduction are likely immune-component specific.

### Immunosenescence

Of the innate immune variables we examined in yellow mud turtles, we found evidence of immunosenescence in all three (Fig. 2; Table 2). Results from other studies are mixed. For example, larger (presumably older) red-eared sliders exhibited no change in BC compared to smaller individuals [32, 61], similar to findings for bactericidal competence in painted turtles where age was known [5]. This latter study also reported increases in haemagglutination in older animals. Obviously, no pattern of age-related changes in the innate immune system in turtles is yet evident. Among other non-avian reptiles, the results are also quite variable (reviewed in [8, 26]). For example in populations of garter snakes, both haemagglutination and haemolysis ability decrease in older snakes [27, 80], as does immune response to the mitogen phytohaemagglutinin in Dickerson’s collared lizards [81]. While in water pythons, haemagglutination increases with age [82]. Further research will be necessary to understand this variation in the direction of age-related changes in innate immunity in reptiles generally, and turtles specifically. In contrast, data from mammals and birds broadly show declines in immune function with age. Furthermore, in these taxa most immunosenescence observed occurred in the adaptive immune system [83–85] with less pronounced changes found in innate immunity (reviewed in [6]), although here too, patterns are not necessarily consistent across innate immunity measures [86]. However, in reptiles, the adaptive immune system responds more slowly to challenges and does not exhibit robust responses during repeated pathogen exposure (reviewed in [24, 87]). Thus, innate immune function, and age-related changes in such function may be critical to overall immune health in reptiles.

## Conclusions

Here we have attempted to expand the comparative considerations of innate immunosenescence, and its interactions with mortality and reproductive ageing. We chose a long-studied wild population of (yellow mud) turtles to test for associations among these measures of ageing because recent broad comparative studies have identified the turtle clade as having exceptionally slow ageing and long lifespans. Notwithstanding, measures of ageing mechanisms require known ages, which is only achievable through long-term study for wild, long-lived species. We found that mortality and immunity had sex-specific characteristics; females lived longer and had lower innate immune function than males. This result is striking based on the temperature-dependency of sex determination, thus ruling out contributions of sex chromosomes to sex-specific ageing. All three measures of innate immunity declined with advancing age. In females, clutch mass increased with advancing age in females, and reproductive output did not predict innate immune function other than that females with larger clutches had higher bactericidal competence, suggesting no obvious cost of reproductive effort for innate immunity in females. This is the first study to examine three axes of ageing in turtles, a group characterized by slow-to-negligible ageing. These findings on immunosenescence, mortality, and reproduction suggest turtles as a new model for understanding the mechanisms of slow ageing, or, more precisely, the study of universal ageing mechanisms (such as immunosenescence) in a monophyletic group characterized by slow adult mortality acceleration and negligible reproductive senescence.

## Methods

### Field sampling

Tissue sampling for the yellow mud turtles (YMT) for this study took place in May 2018 on Gimlet Lake at our long-term research site on the Crescent Lake National Wildlife Refuge (CLNWR), in Garden County, Nebraska, USA (41°45.24′N, 102°26.12′W). The Gimlet Lake marsh complex is a shallow (average depth 0.8 m), sandhill lake with marsh habitat [88]. YMTs exhibit temperature-dependent sex determination (TSD) with females produced under warm incubation conditions and both males and females produced under cooler conditions [59]. Mark-recapture and nesting ecology studies were ongoing here from 1981 through 2018. At this site, YMTs typically overwinter terrestrially buried in upland sandhills adjacent to wetlands, emerge in April and May, and migrate to the water, and then most females return to the same sandhills to nest in June, although some do not reproduce every year [67, 87]. By July all turtles begin leaving the wetlands to estivate in the sandhills for the remainder of the summer (see also [89]). During field seasons, drift fences were constructed parallel to the shore between three overwintering sites and the lake, and monitored continuously each day.

During years (including 2017) when the fences were in place during the nesting season, each captured female was x-rayed to determine clutch size, and the width of each egg on each x-ray was measured. A regression equation relating mean clutch x-ray width with actual mean egg mass from a subset of nests that were subsequently excavated allowed us to estimate egg mass and clutch mass (both in g) for each gravid female in 2017 (*n* = 85). The equations for these relationships and fit are as follows: Actual Egg Width = 0.98(Estimated Egg Width) + 1.52, *R*^2^ = 0.89; Actual Egg Mass = 0.64(Estimated Egg Mass) – 6.07, *R*^2^ = 0.85; and in a sample of *N* = 1795 YMT eggs collected over several years egg width can be reliably used to estimate egg mass (and therefore total clutch mass) Egg Mass = 0.68(Egg Width) – 6.77, *R*^2^ = 0.85 (Iverson, unpublished data). This enabled us to examine the effects of those measures of reproductive output on immunity in the spring of 2018 as these 85 female turtles emerged from brumation.

Once captured, turtles were transported back to the field laboratory where morphometric data were recorded, including maximum carapace length (CL in mm), maximum plastron length (PL in mm), and body mass (BM in g). Up to 0.5 ml of whole blood was collected from the cervical sinus via 26 gauge heparinized syringe, centrifuged (7000 rpm) in a cryotube for 5 min to separate blood components. Blood plasma was pipetted to a separate cryotube. Both plasma and packed red blood cells were immediately flash frozen in liquid nitrogen until transport to Iowa State University for storage at − 80 °C. Sampled turtles (98 males and 102 females) were transported back to their initial capture location and released on the opposite side of the fence to proceed to the lake.

### Age determination

Since this study began in 1981, age in years upon initial capture of each turtle was estimated as the number of winters post-hatching. Age estimation is the same every year and, thus, our methods for age determination apply to all data considered herein (population and immune subsample). Because only a single scute annulus is produced each year in this population, age of immature turtles usually equals the total number of annuli present. However, during extremely harsh years, there may not be any growth, and two annuli may appear as one. Hence, counts of annuli are minimum ages, and might be underestimated if annuli are not counted and evaluated in the context of general shell growth patterns in the whole population. By comparing the pattern of the increments in scute growth of an unaged juvenile turtle with the general pattern of scute growth in the population, age-at-first-capture could be reliably determined up to at least 12 years. We counted the minimum number of annuli on adult turtles captured in the early years of the study, and age was estimated for all of those turtles if the number of annuli was less than 20. However, most turtles were initially marked individually and aged before their sixth winter, and aged subsequently based on the actual recapture interval. Adult females were significantly older (and smaller) than males in our samples (Table S3; unpaired t-test, *P* = 0.0003, See Fig. S2 and Fig. 5 in [33]). Therefore age was z-transformed within each sex to a mean of 0 with unit variance. This variable “zAge” was used in all statistical analyses. We excluded data from 26 unsexed juveniles.

### Reproductive analysis

Reproductive output – clutch size, total clutch mass – increased with increasing size and age. Therefore, for the analyses that included female reproductive output along with age in the model, we used raw values of reproductive output without correcting for body size. All linear models were performed in SAS v. 9.4 (SAS Institute, Cary, NC, USA).

### Survival analysis

Survivorship and age-specific mortality rates of adult *Kinosternon flavescens* from western Nebraska were estimated based on the Gompertz model (Table 1) [18, 90, 91] which estimates an initial mortality probability at starting age, and the rate of accelerating mortality across the lifespan. More complicated models (e.g., including a constant Makeham term for age-independent mortality, including a deceleration parameter) were not supported by these data based on the change in AIC (computed in the *BaSTA* R package [90]). Gompertz modeling was applied to our long-term mark-recapture data set from Gimlet Lake, including data from 1530 individual females and 860 individual males. Age at maturity averaged 11 years for both sexes, which is known from the long-term monitoring of age and reproduction in this population [88, 92, 93]. Because maturation is size-dependent, these estimates of maturation age (i.e., 11 yr.) are maximum estimates (for example, an occasional male turtle can be identified at younger ages). We tested the sensitivity of using an average maturation age of 10 years with no appreciable effect on estimates of lifespan and mortality ageing (data not shown). Maximum and median adult lifespan were calculated as the number of years after the age of first reproduction until 95 and 50% of the adults in the synthetic cohort were estimated to have died. Datasets were analyzed using the ‘basta’ function from the *BaSTA* package for R [90].

### Immune measures

Natural antibody-mediated haemagglutination and complement-mediated haemolysis (NAbs, Lysis) ability are reported to be the first line of defense against pathogens in vertebrates (reviewed in [94]), and these measures of innate immune function have been studied in many reptile species (e.g., [4, 5, 32, 80, 82, 95]). We completed the assays for NAbs and lysis ability according to the haemolysis–haemagglutination assay adapted from [96] for use in painted turtles [5, 97]) using rabbit red blood cells. We used two bottles of rabbit red blood cells (HemoStat HemoStat Laboratories, Dixon, CA, USA) to complete all assays. We ran all plates with positive and negative controls and samples in duplicate. Higher titres for haemagglutination indicate greater abundance of natural antibodies in the plasma sample, as high titres are an indication that natural antibodies are at high concentrations even in increasingly diluted plasma [96]. Similarly, higher titres for haemolysis indicate the plasma is able to lyse RRBCs even at more dilute concentrations [96]. Thus, increased natural antibody levels and lysis ability are associated with increased immune function. We assessed bactericidal competence (BC) of plasma by quantifying its ability to inhibit growth of *Escherichia coli* using our published protocol for painted turtles [5, 97], adapted from [98]. Five lyophilized pellets of *E. coli* (Microbiologics, ATCC#8739) were used in the present experiment, with each new pellet used to generate a new control solution as we progressed through samples. Increased bactericidal competence corresponds to increased immune function. All immune assays were conducted in spring of 2019 on samples collected in spring of 2018.

### Immune measure: statistical analyses

Dependent variables were analyzed using general linear models with explanatory variables of: zAge (standardized age within each sex); Sex (adult males and adult females); the two-way interaction between zAge and Sex; and the nuisance variable assay batch (BC: *N* = 4; NAbs and Lysis: *N* = 2) to account for control pellets (BC) or bottle of rabbit red blood cells (NAbs and Lysis). Bactericidal Competence was Arcsine transformed to account for proportional data and to meet conditions of normality. NAbs and Lysis were log-10 transformed to meet conditions of normality. All linear models were performed in SAS v. 9.4 (SAS Institute, Cary, NC, USA). Graphs were made in ggplot2 [99].

## Supplementary Information

**Additional file 1.**

## Data Availability

The data from this study are available on DataShare: the Open Data Repository of Iowa State University (10.25380/iastate.132220xxx).
