# Supplementary material for: Sex-specific innate immunity and ageing in long-lived fresh water turtles (Kinosternon flavescens: Kinosternidae)
Source: Immun Ageing. 2023 Mar 9;20:11. doi: 10.1186/s12979-023-00335-x (PMC9997018; doi:10.1186/s12979-023-00335-x)

Table S1. Correlation coefficients for condition, age, and immune variables for adult male and female Yellow Mud Turtles in western Nebraska. zAge is z-transformed age; Asin(BC) is Arcsine-transformed Bactericidal Competence; Log(NAbs) is log_{10} Agglutination titre; Log(Lys) is log_{10} Lysis titre. See text for details.

		Asin(BC)	Log(NAbs)	Log(Lys)
**zAge**				
	r	-0.21	-0.17	-0.13
	P_r=0	**0.003**	**0.017**	**0.06**
	N	197	198	198
**Asin(BC)**				
	r		0.22	0.25
	P_r=0		**0.001**	**0.0003**
	N		198	198
**Log(NAbs)**				
	r			0.28
	P_r=0			**0.0001**
	N			199

Table S2. Correlation coefficients for age, size, and reproductive variables for female *Kinosternon flavescens* in western Nebraska that reproduced in 2017.

	BM	Clutch Size	Egg Mass	Clutch Mass	CM/BM
Age					
r	0.74	0.15	0.65	0.42	-0.22
P_r=0	**<0.0001**	0.17	**<0.0001**	**<0.0001**	**0.05**
N	84	85	85	85	84
Body mass (BM)		0.42	0.71	0.68	-0.17
		**<0.0001**	**<0.0001**	**<0.0001**	0.12
		84	84	84	84
Clutch Size					
r			0.23	0.89	0.73
P_r=0			**0.04**	**<0.0001**	**<0.0001**
N			85	85	84
Egg Mass					
r				0.65	0.09
P_r=0				**<0.0001**	0.39
N				85	84
Clutch Mass					
r					0.61
P_r=0					**<0.0001**
N					84

Fig. S1. Relationship between clutch size and bactericidal competence (BC) for female Kinosternon flavescens in western Nebraska that reproduced in 2017. The Y-axis is residuals from the full model in Table 3 excluding clutch size. See Table 3 for statistics.

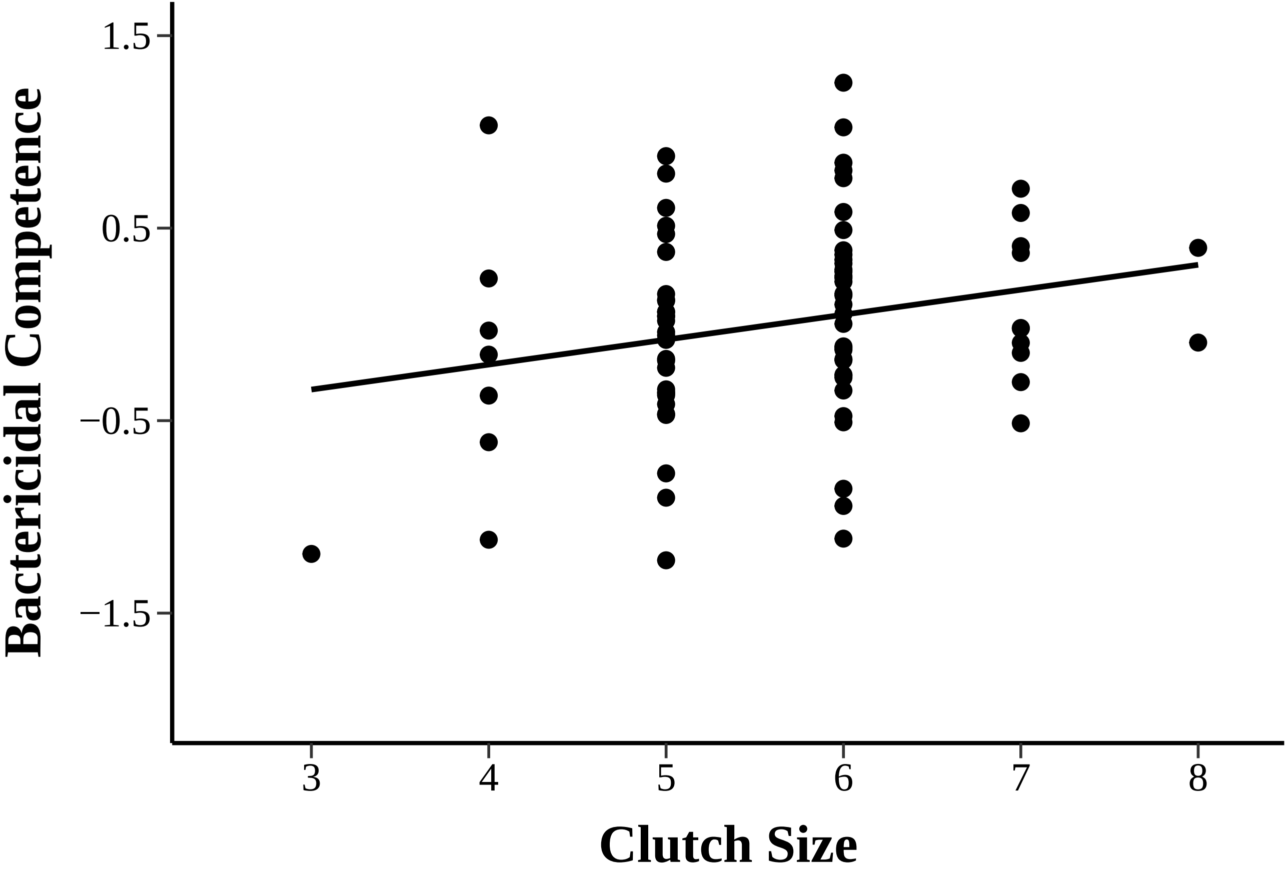

Fig S2. Age distribution of adult male and female yellow mud turtles in this study. Median female and male age significantly differed, and standardized age was used in analyses that included both sexes. See main text for details.

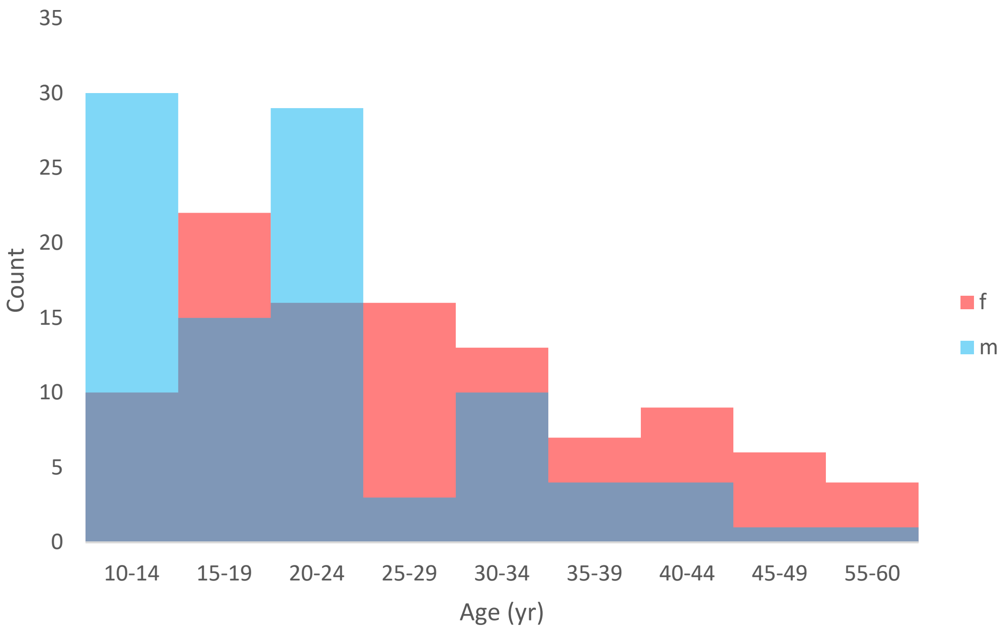

Supplement: Supplementary file 1 — Additional file 1. [file 12979_2023_335_MOESM1_ESM.pdf]
